# NOMA-Assisted Multiple Access Scheme for IoT Deployment: Relay Selection Model and Secrecy Performance Improvement

**DOI:** 10.3390/s19030736

**Published:** 2019-02-12

**Authors:** Dinh-Thuan Do, Minh-Sang Van Nguyen, Thi-Anh Hoang, Miroslav Voznak

**Affiliations:** 1 Wireless Communications Research Group, Faculty of Electrical and Electronics Engineering, Ton Duc Thang University, Ho Chi Minh City, Vietnam; 2 Industrial University of Ho Chi Minh City (IUH), Ho Chi Minh City, Vietnam; nguyenvanminhsang@iuh.edu.vn (M.-S.V.N.); hoangthianh@iuh.edu.vn (T.-A.H.); 3 Department of Telecommunications, VSB—Technical University of Ostrava, 708 00 Ostrava, Czech Republic; miroslav.voznak@vsb.cz

**Keywords:** relay selection, NOMA, IoT, secure outage probability, strictly positive secure capacity

## Abstract

In this paper, an Internet-of-Things (IoT) system containing a relay selection is studied as employing an emerging multiple access scheme, namely non-orthogonal multiple access (NOMA). This paper proposes a new scheme to consider secure performance, to be called relay selection NOMA (RS-NOMA). In particular, we consider metrics to evaluate secure performance in such an RS-NOMA system where a base station (master node in IoT) sends confidential messages to two main sensors (so-called NOMA users) under the influence of an external eavesdropper. In the proposed IoT scheme, both two NOMA sensors and an illegal sensor are served with different levels of allocated power at the base station. It is noticed that such RS-NOMA operates in two hop transmission of the relaying system. We formulate the closed-form expressions of secure outage probability (SOP) and the strictly positive secure capacity (SPSC) to examine the secrecy performance under controlling setting parameters such as transmit signal-to-noise ratio (SNR), the number of selected relays, channel gains, and threshold rates. The different performance is illustrated as performing comparisons between NOMA and orthogonal multiple access (OMA). Finally, the advantage of NOMA in secure performance over orthogonal multiple access (OMA) is confirmed both analytically and numerically.

## 1. Introduction

Any eavesdropper is able to disturb the signal easily due to the broadcasting environment of wireless communication. At the application layer (i.e., highest layer), encryption methodology using cryptography is conventionally implemented to assurance the secure information transmission. Nevertheless, to tackle with situation of speedy growth of computer networks, these procedures and secure keys become ineffective ways, especially in increasing computing capability [1]. Additionally, great encounters in secure communications include the security of key transmission, the complexity of key management, and distribution [2]. Consequently, physical layer security (PLS) is an effective way to fight eavesdropping and diminish the overhearing information and it is considered as an extra data fostering key encryption technology as in [3,4].

To provide a network access technique for the next generation of wireless communications, an emerging multiple access scheme, namely, non-orthogonal multiple access (NOMA) transmission was proposed in many works such as [5]. The power domain and channel quality are acquired to exploit different performance of NOMA users regarding multiple access. As a main characterization, a significantly strengthened performance results from NOMA users with good channels, while relatively poor performance is seen in NOMA users with bad channel conditions [6]. Combining NOMA with cooperative communication [7,8,9], cooperative NOMA (C-NOMA) transmission scheme is proposed as a possible solution to generate a unique system in which users with better channel circumstances assist forwarding signal to distance users who are affected in situations of worse channels [7,10].

To achieve an advantage of the diversity related to wireless channels in relaying networks, a relay selection scheme has been broadly implemented and considered as improving the quality of the transmission [11]. Especially, a relay network is introduced in some technical deployment snapshots of the IoT devices of SmartBridge, SmartDIMES, and SmartSenSysCalLab [12]. Two policies in energy harvesting architecture inclusing time switching (TS) relaying, power splitting (PS) relaying are empoyed with NOMA and it is considered as suitable deployment of wireless powered IoT relay systems [13]. In a practical scenario, main technologies for wireless communication systems (for example LTE) are required to deploy multiuser selection or scheduling schemes. In addition, the relay selection scheme under NOMA networks is introduced and analysed in recent works [14,15,16]. A great improvement in the QoS of the system is resulted from a system model which combines cooperative relay and NOMA. In particular, a two-stage relay selection is proposed and derived with respect to closed-form expressions on outage probability and they are obtained in cooperative systems using decode-and-forward as in [14]. The approximate and asymptotic expressions on average sum rate are examined as combining relay selection and amplify-and-forward (AF) assisted NOMA [15]. Moreover, by analyzing the outage probability and its asymptotic results, a partial relay selection scheme is studied in [16]. The fixed and adaptive power allocations (PAs) at the relays are introduced in cooperative NOMA to consider two optimal relay selection schemes, namely as the two-stage weighted-max-min (WMM) and max-weighted-harmonic-mean (MWHM) schemes [17]. On the orther hand, to improve the performance in throughput and coverage, new model is exploited as combining the orthogonal frequency division multiple access (OFDMA) and cooperative multicast (CM) technology to perform the intra-cooperation of multicast group (MG) [18]. In other systems, relay selection (RS) non-orthogonal multiple access (NOMA) is studied in terms of the diversity orders by deployment of RS schemes for full-duplex /half-duplex communications [19].

Furthermore, power allocation and user scheduling are discussed as the other encounters in NOMA networks [20]. To improve the NOMA’s performance, power distribution therein shows a major characterization affecting different user’s performance since certain power partitions which are allocated for multiple superposed users, and this topic fascinates a lot of study. For instance, fixed power allocation scheme is deploy to serve two NOMA users and its performance is evaluated by employing the closed-form expression of outage probability and ergodic sum-rate in [21]. In addition, a general two-user power allocation algorithm is proposed by overcoming the drawbacks of fixed power distribution in NOMA network [22]. On the other hand, fairness performance of NOMA network is resulted by varying power allocation factors as investigation in [23]. While sum rate maximization and proportional fairness criteria under impact of the power allocation algorithms is studied for two user NOMA networks in [24].

On the other hand, stochastic geometry networks are exploited regarding the physical layer security to apply to 5G NOMA networks in [25]. To enhance the secrecy performance for single antenna and multiple-antenna stochastic geometry networks two dissimilar schemes were considered as extended work of [25] and detailed contribution can be observed in [26]. Furthermore, the optimal decoding order, power allocation and transmission rates are important metrics to evaluate and exhibit a new design of NOMA under secrecy considerations [27]. A single-input single-output (SISO) system serving NOMA scheme was investigated in terms of secure performance in [28]. In such system, optimal power allocation policy is proposed to highlight advantage of secrecy performance of NOMA compared with that in the conventional OMA [28]. The authors in [29] exploited physical layer security in downlink of NOMA systems [29] and both the exact and asymptotic secrecy outage probability (SOP) were investigated to examine secure performance of the SISO and MISO NOMA systems. In other trend of research, two transmit antenna selection (TAS) schemes were proposed to perform secure performance evaluation in cooperative NOMA networks in [30], and then the closed-form formula of the ergodic secrecy rate was achieved. To the best of the authors’ knowledge, there are few works related to the analysis of the physical layer security in relay selection NOMA systems. Thus, this is the main motivation of this work.

From the above analysis, it is worth noting that a few studies have considered the technical design of NOMA relaying architecture against the unwanted eavesdropper with appropriate secrecy. This paper aims to exploit the advantage of relay selection to improve system performance of IoT deploying NOMA. In particular, this motivates us to design secure NOMA schemes for the practical IoT scenario where the relay is selected to forward signal with enhanced performance at NOMA receivers. In this scenario, we use the secrecy probability to measure the secrecy performance of the system since the perfect secrecy rate is usually not obtained, and hence, it can not be evaluated as the secrecy metric. We highlight that the SOP and SPSC are appropriate secrecy metrics for security consideration in the NOMA systems.

The primary contributions of the paper are summarized as follows:

- Targeting the secrecy outage constraint, we comprehensively study the design of NOMA-assisted IoT system against the external eavesdropper. The transmit signal to noise ratio (SNR) at the base station (BS), transmission rates, and power allocated factors to each user are considered as main parameters. These values need be determined in design of RS-NOMA. For the first time, we analytically prove that the relay selection provides improved secure performance at higher number of relay for RS-NOMA.
- For Decode-and-Forward (DF) mode, we show that the outage behavior of RS- NOMA scheme is superior to that of OMA scheme in the specific SNR region. Furthermore, we confirm that the RS-NOMA scheme depends on how strong the eavesdropper channel is. In fact, SOP and SPSC of far user depend on the number of relay selected.
- Both analytically and numerically, the exactness of derived expressions is verified and we compare the performance of the NOMA scheme with that of the OMA scheme in the studied problems with the secrecy outage constraint.

The remainder of this paper is organized as follows. In Section 2, the system model is introduced. The detailed analysis in terms of SOP metric is proposed in Section 3. In Section 4, we derive an exact expression of SPSC in RS-NOMA. Section 5 presents the benchmark of OMA scheme for further evaluation. Numerical results are presented in Section 6. Concluding remarks are given in Section 7.

The main notations of this paper are shown as follows: $E\left\{\cdot \right\}$ denotes expectation operation; $f_{X}\left(.\right)$ and $F_{X}\left(.\right)$ stand for the probability density function (PDF) and the cumulative distribution function (CDF) of a random variable *X*.

## 2. System Model of Secure Analysis for DF Relay Selection

**Figure 1:**
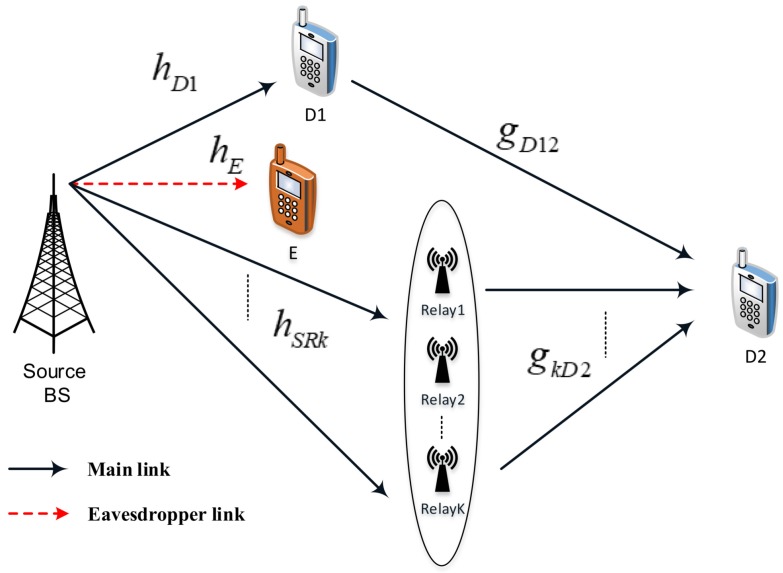
System model of a RS-NOMA assisted IoT system in the existence of an external eavesdropper.

Figure 1 represents the considered RS-NOMA assisted IoT system including a base station (BS), multiple relays (i.e., *K* relays), two main sensors (D1, strong user, and D2, poor user), and an eavesdropper (E) in an IoT network. In such a system model, the BS is located in the cell-center, strong user D1 and E are located near with the BS while the poor user D2 is very close to the cell-edge. In this situation, it is assumed that there is no direct links between BS and the poor user due to high obstructions or deep fading. However, quality of transmission from the BS to D2 will be improved by employing relay selection scheme. We further assume that single antenna is equipped at all nodes in the RS-NOMA network and each link employing channels associated with independent Rayleigh fading. As most expectations in the literature, it is assumed that E can acquire the signals transmitted from the BS.

The channel coefficients from the BS to relay k,k=1,2,…,K and the eavesdropper are denoted by $h_{SRk}$ and $h_{E}$, respectively. Next, the channel coefficient from the BS to near NOMA user is $h_{D1}$, while $g_{kD2}$ is denoted as channel coefficient between relay *k* and D2. These channels are normalized as Rayleigh fading channel. We assume the quasi-static block fading model adopted; it means the channel coefficients are kept constant during the transmission of one message, which includes a block of symbols, and adjust independently of one block to the next block. We call $P_{S}$ is transmit power at the BS, $\alpha_{1}$, $\alpha_{2}$ are power allocation factors for two NOMA users and they satisfy $\alpha_{1}+\alpha_{2}=1$. It is noted that $x_{1}$, $x_{2}$ are simultaneous transmissions from the BS to serve two NOMA users D1, D2 respectively. In addition, we denote $w_{U}$ as Additive white Gaussian noise (AWGN) term at node *U*.

As a fundamental principle of RS-NOMA, the transmitter is enabled to simultaneously assist multiple users. To perform this task, the superposition coding (SC) is deployed in the transmitter to conduct a linear combination of multiple signals to serve the users. The composed signal $x_{S}^{NOMA}$ is transmitted from the BS to all relays and two NOMA users in the first phase, which is shown as

(1) $$x_{S}^{NOMA}=\sqrt{\alpha_{1}P_{S}}x_{1}+\sqrt{\alpha_{2}P_{S}}x_{2}.$$

The received signal at D1 in the direct link is expressed by

(2) $$\begin{array}{cc} y_{SD1}^{NOMA} & =h_{D1}x_{S}^{NOMA}+w_{D1}\\ & =h_{D1}\left(\sqrt{\alpha_{1}P_{S}}x_{1}+\sqrt{\alpha_{2}P_{S}}x_{2}\right)+w_{D1}. \end{array}$$

Here, it is AWGN noise and variance of $\sigma_{0}^{2}$.

The received signal at $R_{k}$ is given by

(3) $$\begin{array}{cc} y_{SRk}^{NOMA} & =h_{SRk}x_{S}^{NOMA}+w_{R}\\ & =h_{SRk}\left(\sqrt{\alpha_{1}P_{S}}x_{1}+\sqrt{\alpha_{2}P_{S}}x_{2}\right)+w_{Rk}. \end{array}$$

In this paper, it is assumed that users are not arranged by their channel conditions. Under such considered RS-NOMA scheme, $x_{2}$ can be detected at user 1 before using successive interference cancellation (SIC) [6]. Therefore, the received instantaneous signal-to-interference-noise ratio (SINR) of the user D1 can be given as SNR to detect $x_{2}$ as

(4) $$\begin{array}{c} \gamma_{SD1,x2}^{NOMA}=\frac{\alpha_{2}P_{S}{\left|h_{D1}\right|}^{2}}{\alpha_{1}P_{S}{\left|h_{D1}\right|}^{2}+\sigma_{0}^{2}}=\frac{\alpha_{2}\rho_{S}{\left|h_{D1}\right|}^{2}}{\alpha_{1}\rho_{S}{\left|h_{D1}\right|}^{2}+1}. \end{array}$$

The SIC is carried out at D1 to remove the signal for D2, therefore the instantaneous rate for D1 detect the signal $x_{1}$ is given by

(5) $$\begin{array}{c} \gamma_{SD1,x1}^{NOMA}=\frac{\alpha_{1}P_{S}{\left|h_{D1}\right|}^{2}}{\sigma_{0}^{2}}=\alpha_{1}\rho_{S}{\left|h_{D1}\right|}^{2} \end{array}$$

where $\rho_{S}=\frac{P_{S}}{\sigma_{0}^{2}}$.

In this situation, it is possible to apply fixed power allocation coefficients in two NOMA users in such relay selection mode. To improve the performance of the relay selection schemes, reasonable power optimization can be further studied, and this concern may be considered in our future work.

At relay, $x_{2}$ can be detected before using SIC and as employing SIC, $x_{2}$ will be regarded as interference eliminated before decoding signal $x_{1}$. It is assumed that these relays can not harm D1 and there is no detection on $x_{1}$. Firstly, the expression of SNR must be computed to decode $x_{2}$ transmitted from the BS to relay as

(6) $$\begin{array}{c} \gamma_{SR,x2}^{NOMA}=\frac{\alpha_{2}P_{S}{\left|h_{SRk}\right|}^{2}}{\alpha_{1}P_{S}{\left|h_{SRk}\right|}^{2}+\sigma_{0}^{2}}=\frac{\alpha_{2}\rho_{S}{\left|h_{SRk}\right|}^{2}}{\alpha_{1}\rho_{S}{\left|h_{SRk}\right|}^{2}+1}. \end{array}$$

At the cell-edge user, the received signal can be obtained at D2 from the relay as

(7) $$\begin{array}{c} y_{RD_{2}}^{NOMA}=g_{kD2}\sqrt{P_{R}}x_{2}+w_{D_{2}}. \end{array}$$

Therefore, calculating SNR to detect $x_{2}$, which is transmitted in the second hop from the *k*th relay to user D2, is given as

(8) $$\begin{array}{c} \gamma_{RD2,x2}^{NOMA}=\frac{P_{R}{\left|g_{kD2}\right|}^{2}}{\sigma_{0}^{2}}=\rho_{R}{\left|g_{kD2}\right|}^{2}, \end{array}$$

where $\rho_{R}=\frac{P_{R}}{\sigma_{0}^{2}}$.

The received signal at D2 which forwarded by D1 is expressed as

(9) $$\begin{array}{c} y_{D1D2}^{NOMA}=g_{D12}\sqrt{P_{R}}x_{2}+w_{D_{2}}. \end{array}$$

The received SINR at D2 to get $x_{2}$ for link is given by

(10) $$\begin{array}{c} \gamma_{D12,x2}^{NOMA}=\frac{P_{R}{\left|g_{D12}\right|}^{2}}{\sigma_{0}^{2}}=\rho_{R}{\left|g_{D12}\right|}^{2}. \end{array}$$

Regarding computation of the received signal to interference plus noise ratio (SINRs) at the eavesdropper, here, we overestimate the eavesdropper’s capability. A worst-case assumption from the legitimate user’s perspective is made here. That is, *E* is equipped capability of the multiuser detection. In more detailed consideration, user E performs parallel interference cancellation (PIC) to distinguish the superimposed mixture. In such a scenario, the eavesdropper knows the decoding order and the power allocation factors. Thus, we have to adopt the worst-case assumption from the legitimate user’s perspective due to the conservativeness mandated by the security studies. It is worth noting that this assumption has been adopted in previous work on the secrecy of NOMA systems [25,26]. It is shown that the received signal at *E* is

(11) $$\begin{array}{cc} y_{SE}^{NOMA} & =h_{E}x_{S}^{NOMA}+w_{E}\\ & =h_{E}\left(\sqrt{\alpha_{1}P_{S}}x_{1}+\sqrt{\alpha_{2}P_{S}}x_{2}\right)+w_{E}. \end{array}$$

Therefore, SNR is computed to overhear $x_{1}$ at *E* as

(12) $$\begin{array}{c} \gamma_{SE1}^{NOMA}=\frac{\alpha_{1}P_{S}{\left|h_{E}\right|}^{2}}{\sigma_{E}^{2}}=\alpha_{1}\rho_{E}{\left|h_{E}\right|}^{2}, \end{array}$$

where $\rho_{E}=\frac{P_{S}}{\sigma_{E}^{2}}$, Here, AWGN noise term at E has variance of $\sigma_{E}^{2}$.

And then, SNR related to overhearing signal $x_{2}$ at E is given by

(13) $$\begin{array}{c} \gamma_{SE2}^{NOMA}=\frac{\alpha_{2}P_{S}{\left|h_{E}\right|}^{2}}{\sigma_{E}^{2}}=\alpha_{2}\rho_{E}{\left|h_{E}\right|}^{2}. \end{array}$$

In this RS-NOMA scheme, the best relay node is selected by the following criterion. Firstly, the end-to-end SNR following DF mode can be computed by [31]

(14) $$\begin{array}{c} \gamma_{k}^{NOMA}=\min\left(\gamma_{SRk,x2}^{NOMA},\gamma_{RD2,x2}^{NOMA}\right), \end{array}$$

where $\gamma_{SRk,x2}$ stands for SNR at the first hop from the BS transmitting signal to the *k*th relay Rk.

The index $k^{*}$ in group of relay in considered criteria is determined by

(15) $$\begin{array}{c} \gamma_{k*}^{NOMA}=\max_{k=1,\ldots ,K}\left(\gamma_{k}^{NOMA}\right) \end{array}.$$

The secrecy capacity for D1 is obtained as

(16) $$\begin{array}{c} C_{x1}^{NOMA}={\left[\frac{1}{2}\log_{2}\left(\frac{1+\min\left(\gamma_{SD1,x1}^{NOMA},\gamma_{SD1,x2}^{NOMA}\right)}{1+\gamma_{SE1}^{NOMA}}\right)\right]}^{+}, \end{array}$$

where ${\left[x\right]}^{+}=\max\left\{x,0\right\}$. It is worth noting that D2 employs Maximum ratio combining (MRC) principle to process mixture signal as existence of both D1-D2 link and Source-Selected Relay-D2 link. As a result, the secrecy capacity for D2 is obtained as [16]

(17) $$\begin{array}{c} C_{x2}^{NOMA}={\left[\frac{1}{2}\log_{2}\left(\frac{1+\max\left(\min\left(\gamma_{SD1,x2}^{NOMA},\gamma_{D12,x2}^{NOMA}\right),\gamma_{k*}^{NOMA}\right)}{1+\gamma_{SE2}^{NOMA}}\right)\right]}^{+}. \end{array}$$

## 3. Secure Outage Performance in RS-NOMA

In this section, the secrecy capacity is studied for Rayleigh fading channels in terms of the SOP. To describe the secrecy performance of a wireless communication system, such a metric is also an important performance measurement and SOP is generally used. In particular, the SOP is defined as the probability that the instantaneous secrecy capacity $C_{sec}$ will drop below a required secrecy rate threshold *R* (i.e., if $C_{sec}<R$, information security will not be satisfied, and then an outage event can be raised; otherwise, perfect secrecy will be maintained).

### 3.1. SOP at D1

**Proposition** **1.** *The SOP for D1 can be expressed as*

(18) $$\begin{array}{c} P_{SOP1}^{NOMA}=1-\frac{\alpha_{1}\rho_{S}\lambda_{D1}}{\left(\alpha_{1}\rho_{S}\lambda_{D1}+\varphi_{1}\lambda_{E}\right)\varphi_{1}\alpha_{1}\lambda_{E}}\exp\left(-\frac{\psi_{1}}{\alpha_{1}\rho_{S}\lambda_{D1}}+\frac{1}{\alpha_{1}\rho_{S}\lambda_{D1}}-\frac{\alpha_{2}-\psi_{1}\alpha_{1}}{\varphi_{1}\alpha_{1}\lambda_{E}}\right)U\left(t_{1}\right), \end{array}$$

*where $\varphi_{1}=2^{2R_{1}}\alpha_{1}\rho_{E},\psi_{1}=2^{2R_{1}}-1,U\left(t_{1}\right)=\int_{0}^{\alpha_{2}-\psi_{1}\alpha_{1}}\exp\left(-\frac{\alpha_{2}}{\alpha_{1}t_{1}\rho_{S}\lambda_{D1}}+\frac{t_{1}}{\varphi_{1}\alpha_{1}\alpha_{1}\rho_{S}\lambda_{E}}\right)dt_{1}$. From here to following sections, we denote $\lambda_{D1}$, $\lambda_{D12}$, $\lambda_{SRk}$, $\lambda_{kD2}$, $\lambda_{E}$ as channel gains of links BS-D1, D1-D2, BS-Rk, Rk-D2, BS-E respectively. Here, $R_{1}$ denotes the target data rate of D1.*

**Proof.**  See in Appendix A. ☐

### 3.2. SOP at D2

**Proposition** **2.** *For performance evaluation on user D2, we formulate SOP as*

(19) $$\begin{array}{cc} P_{SOP2}^{NOMA} & =1-\frac{\rho_{R}\lambda_{D12}}{\left(\rho_{R}\lambda_{D12}+\varphi_{2}\lambda_{E}\right)\varphi_{2}\alpha_{1}\rho_{S}\lambda_{E}}\exp\left(\frac{1}{\alpha_{1}\rho_{S}\lambda_{D1}}-\frac{\alpha_{2}\rho_{S}-\psi_{2}\alpha_{1}\rho_{S}}{\varphi_{2}\alpha_{1}\rho_{S}\lambda_{E}}-\frac{\psi_{2}}{\rho_{R}\lambda_{D12}}\right)q\left(t_{2}\right)\\ & \times \prod_{k=1}^{K}\left(\frac{\rho_{R}\lambda_{kD2}}{\left(\rho_{R}\lambda_{kD2}+\varphi_{2}\lambda_{E}\right)\varphi_{2}\alpha_{1}\lambda_{E}}\exp\left(\frac{1}{\alpha_{1}\rho_{S}\lambda_{SR1}}-\frac{\alpha_{2}-\psi_{2}\alpha_{1}}{\varphi_{2}\alpha_{1}\lambda_{E}}-\frac{\psi_{2}}{\rho_{R}\lambda_{kD2}}\right)q\left(t_{3}\right)\right), \end{array}$$

*where $\varphi_{2}=2^{2R_{2}}\alpha_{2}\rho_{E},\psi_{2}=2^{2R_{2}}-1,q\left(t_{2}\right)=\int_{0}^{\alpha_{2}\rho_{S}-\psi_{2}\alpha_{1}\rho_{S}}\exp\left(-\frac{\alpha_{2}\rho_{S}}{\alpha_{1}\rho_{S}t_{2}\lambda_{D1}}+\frac{t_{2}}{\varphi_{2}\alpha_{1}\rho_{S}\lambda_{E}}\right)dt_{2},q\left(t_{3}\right)=\int_{0}^{\alpha_{2}-\psi_{2}\alpha_{1}}\exp\left(-\frac{\alpha_{2}}{\alpha_{1}t_{3}\rho_{S}\lambda_{SR1}}+\frac{t_{3}}{\varphi_{2}\alpha_{1}\lambda_{E}}\right)dt_{3}$. We denote $R_{2}$ as the target data rate of D2.*

**Proof.**  See in Appendix B. ☐

The secure performance can be examined for the whole NOMA system by deploying this formula

(20) $$\begin{array}{c} OP_{NOMA}=1-\left(1-OP_{1-NOMA}\right)\left(1-OP_{2-NOMA}\right). \end{array}$$

## 4. SPSC Analysis in RS-NOMA

In such RS-NOMA, the SPSC is fundamentally defined as the probability of the secrecy capacity $C_{sec}$ being zero. Under this circumstance, SPSC is an extra metric characterizing the properties of physical channels in wireless communication, and hence, physical-layer (PHY) security is perfectly evaluated to exhibit the RS-NOMA scheme to real application under the existence of eavesdropper in nature wireless transmission environment. In general, the SPSC can be calculated by

(21) $$P_{SPSC}=\mathrm{Pr}\left(C_{sec}>0\right)$$

### 4.1. SPSC Compution at D1

From the definition above, we have the outage formula in this case as

(22) $$\begin{array}{cc} P_{SPSC1}^{NOMA} & =\mathrm{Pr}\left(C_{x1}^{NOMA}>0\right)\\ & =\mathrm{Pr}\left(\gamma_{SD1,x1}^{NOMA}>\gamma_{SE1}^{NOMA},\gamma_{SD1,x2}^{NOMA}>\gamma_{SE1}^{NOMA}\right)\\ & \approx \underset{P_{1}}{\underset{︸}{\mathrm{Pr}\left(\gamma_{SD1,x1}^{NOMA}>\gamma_{SE1}^{NOMA}\right)}}\underset{P_{2}}{\underset{︸}{\mathrm{Pr}\left(\gamma_{SD1,x2}^{NOMA}>\gamma_{SE1}^{NOMA}\right)}}. \end{array}$$

Such outage event must be constrained by $\frac{\rho_{E}{\left|h_{E}\right|}^{2}}{\rho_{S}}>\frac{\alpha_{1}\rho_{E}{\left|h_{E}\right|}^{2}}{\alpha_{2}\rho_{S}-\alpha_{1}\rho_{S}\alpha_{1}\rho_{E}{\left|h_{E}\right|}^{2}}$. Firstly, $P_{1}$ can be written by

(23) $$\begin{array}{cc} P_{1} & =\mathrm{Pr}\left({\left|h_{D1}\right|}^{2}>\frac{\rho_{E}}{\rho_{S}}{\left|h_{E}\right|}^{2}\right)\\ & =\int_{0}^{\frac{\alpha_{2}-\alpha_{1}}{\alpha_{1}\alpha_{1}\rho_{E}}}\exp\left(-\frac{\rho_{E}x}{\rho_{S}\lambda_{D1}}\right)\frac{1}{\lambda_{E}}\exp\left(-\frac{x}{\lambda_{E}}\right)dx\\ & =\frac{\rho_{S}\lambda_{D1}}{\rho_{S}\lambda_{D1}+\rho_{E}\lambda_{E}}\left(\exp\left(-\left(\frac{\rho_{E}}{\rho_{S}\lambda_{D1}}+\frac{1}{\lambda_{E}}\right)\frac{\alpha_{2}-\alpha_{1}}{\alpha_{1}\alpha_{1}\rho_{E}}\right)-1\right) \end{array}$$

Similarly, in case of $\frac{\alpha_{1}\rho_{E}{\left|h_{E}\right|}^{2}}{\alpha_{2}\rho_{S}-\alpha_{1}\rho_{S}\alpha_{1}\rho_{E}{\left|h_{E}\right|}^{2}}>\frac{\rho_{E}{\left|h_{E}\right|}^{2}}{\rho_{S}}$, $P_{2}$ can be calculated as

(24) $$\begin{array}{cc} P_{2} & =\mathrm{Pr}\left({\left|h_{D1}\right|}^{2}>\frac{\alpha_{1}\rho_{E}{\left|h_{E}\right|}^{2}}{\alpha_{2}\rho_{S}-\alpha_{1}\rho_{S}\alpha_{1}\rho_{E}{\left|h_{E}\right|}^{2}}\right)\\ & =\int_{\frac{\alpha_{2}-\alpha_{1}}{\alpha_{1}\alpha_{1}\rho_{E}}}^{\infty }\exp\left(-\frac{\alpha_{1}\rho_{E}x}{\left(\alpha_{2}\rho_{S}-\alpha_{1}\rho_{S}\alpha_{1}\rho_{E}x\right)\lambda_{D1}}\right)\frac{1}{\lambda_{E}}\exp\left(-\frac{x}{\lambda_{E}}\right)dx \end{array}$$

To calculate the above integral, we set new variable as $v=\alpha_{2}\rho_{S}-\alpha_{1}\rho_{S}\alpha_{1}\rho_{E}x\to x=\frac{\alpha_{2}\rho_{S}-v}{\alpha_{1}\rho_{S}\alpha_{1}\rho_{E}}$, then it can be expressed by

(25) $$\begin{array}{cc} P_{2}=\frac{1}{\lambda_{E}} & \int_{\alpha_{1}\rho_{S}}^{\alpha_{2}\rho_{S}}\exp\left(-\frac{\alpha_{2}\rho_{S}-v}{\alpha_{1}\rho_{S}v\lambda_{D1}}-\frac{\alpha_{2}\rho_{S}-v}{\alpha_{1}\rho_{S}\alpha_{1}\rho_{E}\lambda_{E}}\right)\frac{dv}{-\alpha_{1}\rho_{S}\alpha_{1}\rho_{E}}\\ & =\frac{1}{\alpha_{1}\rho_{S}\alpha_{1}\rho_{E}\lambda_{E}}\exp\left(\frac{1}{\alpha_{1}\rho_{S}\lambda_{D1}}-\frac{\alpha_{2}\rho_{S}}{\alpha_{1}\rho_{S}\alpha_{1}\rho_{E}\lambda_{E}}\right)q\left(v\right). \end{array}$$

Therefore, the SPSC is then computed to evaluate secure performance at D1 as

(26) $$\begin{array}{cc} P_{SPSC1}^{NOMA} & =P_{1}\times P_{2}\\ & =m\left(\begin{array}{c} q\left(v\right)\exp\left(-\frac{n\left(\alpha_{2}-\alpha_{1}\right)}{\alpha_{1}\alpha_{1}\rho_{E}}+\frac{1}{\alpha_{1}\rho_{S}\lambda_{D1}}-\frac{\alpha_{2}\rho_{S}}{\alpha_{1}\alpha_{1}\rho_{S}\rho_{E}\lambda_{E}}\right)\\ -q\left(v\right)\exp\left(\frac{1}{\alpha_{1}\rho_{S}\lambda_{D1}}-\frac{\alpha_{2}\rho_{S}}{\alpha_{1}\alpha_{1}\rho_{S}\rho_{E}\lambda_{E}}\right) \end{array}\right), \end{array}$$

where $m=\frac{\rho_{S}\lambda_{D1}}{\left(\rho_{S}\lambda_{D1}+\rho_{E}\lambda_{E}\right)\alpha_{1}\alpha_{1}\rho_{S}\rho_{E}\lambda_{E}},n=\frac{\rho_{E}}{\rho_{S}\lambda_{D1}}+\frac{1}{\lambda_{E}},q\left(v\right)=\int_{\alpha_{2}\rho_{S}}^{\alpha_{1}\rho_{S}}\exp\left(-\frac{\alpha_{2}}{\alpha_{1}v\lambda_{D1}}+\frac{v}{\alpha_{1}\rho_{S}\alpha_{1}\rho_{E}\lambda_{E}}\right)dv$.

### 4.2. SPSC Computation at D2

In a similar way, the SPSC performance at D2 can be expressed as

(27) $$\begin{array}{cc} P_{SPSC2}^{NOMA} & =\mathrm{Pr}\left(C_{x2}^{NOMA}>0\right)\\ & =\underset{G}{\underset{︸}{\mathrm{Pr}\left(\min\left(\gamma_{SD1,x2}^{NOMA},\gamma_{D12,x2}^{NOMA}\right)>\gamma_{SE2}^{NOMA}\right)}}\\ & \times \underset{H}{\underset{︸}{\mathrm{Pr}\left(\max_{k=1\ldots K}\left(\min\left(\gamma_{SR,x2}^{NOMA},\gamma_{RKD2,x2}^{NOMA}\right)\right)>\gamma_{SE2}^{NOMA}\right)}}. \end{array}$$

To proceed from this formula, we first consider term of *G* and it can be calculated as

(28) $$\begin{array}{cc} G & =\mathrm{Pr}\left(\min\left(\gamma_{SD1,x2}^{NOMA},\gamma_{D12,x2}^{NOMA}\right)>\alpha_{2}\rho_{E}{\left|h_{E}\right|}^{2}\right)\\ & =\underset{G_{1}}{\underset{︸}{\mathrm{Pr}\left(\frac{\alpha_{2}\rho_{S}{\left|h_{D1}\right|}^{2}}{\alpha_{1}\rho_{S}{\left|h_{D1}\right|}^{2}+1}>\alpha_{2}\rho_{E}{\left|h_{E}\right|}^{2}\right)}}\underset{G_{2}}{\underset{︸}{\mathrm{Pr}\left(\rho_{R}{\left|g_{D12}\right|}^{2}>\alpha_{2}\rho_{E}{\left|h_{E}\right|}^{2}\right)}}. \end{array}$$

It is worth noting that the outage probability must satisfy the condition of $\rho_{S}-\alpha_{1}\rho_{S}\rho_{E}{\left|h_{E}\right|}^{2}>0\to {\left|h_{E}\right|}^{2}<\frac{1}{\alpha_{1}\rho_{E}}$. As a result, it can be rewritten as

(29) $$\begin{array}{cc} G_{1} & =\int_{0}^{\frac{1}{\alpha_{1}\rho_{E}}}\exp\left(-\frac{\rho_{E}x}{\left(1-\alpha_{1}\rho_{E}x\right)\rho_{S}\lambda_{D1}}\right)\frac{1}{\lambda_{E}}\exp\left(-\frac{x}{\lambda_{E}}\right)dx\\ & =\frac{1}{\lambda_{E}}\int_{0}^{\frac{1}{\alpha_{1}\rho_{E}}}\exp\left(-\frac{\rho_{E}x}{\left(1-\alpha_{1}\rho_{E}x\right)\rho_{S}\lambda_{D1}}-\frac{x}{\lambda_{E}}\right)dx. \end{array}$$

Next, a new variable can be put as $v_{1}=1-\alpha_{1}\rho_{E}x\to x=\frac{1-v_{1}}{\alpha_{1}\rho_{E}}$ to calculate the above integral. As a result, it can be expressed by

(30) $$\begin{array}{cc} G_{1} & =\frac{1}{\lambda_{E}}\int_{1}^{0}\exp\left(-\frac{1-v_{1}}{\alpha_{1}v_{1}\rho_{S}\lambda_{D1}}-\frac{1-v_{1}}{\alpha_{1}\rho_{E}\lambda_{E}}\right)\frac{dv_{1}}{-\alpha_{1}\rho_{E}}\\ & =\frac{1}{\alpha_{1}\rho_{E}\lambda_{E}}\exp\left(\frac{1}{\alpha_{1}\rho_{S}\lambda_{D1}}-\frac{1}{\alpha_{1}\rho_{E}\lambda_{E}}\right)q\left(v_{1}\right), \end{array}$$

where $q\left(v_{1}\right)=\int_{0}^{1}\exp\left(-\frac{1}{\alpha_{1}v_{1}\rho_{S}\lambda_{D1}}+\frac{v_{1}}{\alpha_{1}\rho_{E}\lambda_{E}}\right)dv_{1}$.

Similarly, we have

(31) $$\begin{array}{cc} G_{2} & =\mathrm{Pr}\left({\left|h_{D12}\right|}^{2}>\frac{\alpha_{2}\rho_{E}}{\rho_{R}}{\left|h_{E}\right|}^{2}\right)\\ & =\int_{0}^{\infty }\exp\left(-\frac{\alpha_{2}\rho_{E}x}{\rho_{R}\lambda_{D12}}\right)\frac{1}{\lambda_{E}}\exp\left(-\frac{x}{\lambda_{E}}\right)dx\\ & =\frac{\rho_{R}\lambda_{D12}}{\rho_{R}\lambda_{D12}+\alpha_{2}\rho_{E}\lambda_{E}}. \end{array}$$

From (30) and (31), we have

(32) $$\begin{array}{c} G=\frac{\rho_{R}\lambda_{D12}}{\left(\rho_{R}\lambda_{D12}+\alpha_{2}\rho_{E}\lambda_{E}\right)\alpha_{1}\rho_{E}\lambda_{E}}\exp\left(\frac{1}{\alpha_{1}\rho_{S}\lambda_{D1}}-\frac{1}{\alpha_{1}\rho_{E}\lambda_{E}}\right)q\left(v_{1}\right). \end{array}$$

From (27), *H* can be calculated as

(33) $$\begin{array}{cc} H & =\mathrm{Pr}\left(\max_{k=1\ldots K}\left(\min\left(\gamma_{SR,x2}^{NOMA},\gamma_{RKD2,x2}^{NOMA}\right)\right)>\gamma_{SE2}^{NOMA}\right)\\ & =\prod_{k=1}^{K}\left(\underset{H_{1}}{\underset{︸}{\mathrm{Pr}\left(\frac{\alpha_{2}\rho_{S}{\left|h_{SR1}\right|}^{2}}{\alpha_{1}\rho_{S}{\left|h_{SR1}\right|}^{2}+1}>\zeta \right)}}\underset{H_{2}}{\underset{︸}{\mathrm{Pr}\left(\rho_{R}{\left|g_{kD2}\right|}^{2}>\zeta \right)}}\right). \end{array}$$

$H_{1}$ can be computed as:

(34) $$\begin{array}{cc} H_{1} & =\mathrm{Pr}\left(\frac{\alpha_{2}\rho_{S}{\left|h_{SR1}\right|}^{2}}{\alpha_{1}\rho_{S}{\left|h_{SR1}\right|}^{2}+1}>\zeta \right)\\ & =\mathrm{Pr}\left({\left|h_{SR1}\right|}^{2}>\frac{\zeta }{\left(\alpha_{2}-\zeta \alpha_{1}\right)\rho_{S}}\right)\\ & =\left\{\begin{array}{cc} \exp\left(-\frac{\zeta }{\left(\alpha_{2}-\zeta \alpha_{1}\right)\rho_{S}\lambda_{SR1}}\right), & \zeta <\frac{\alpha_{2}}{\alpha_{1}}\\ 0, & \zeta \geq \frac{\alpha_{2}}{\alpha_{1}} \end{array}.\right. \end{array}$$

Similarly, we can calculate $H_{2}$ to be

(35) $$\begin{array}{c} H_{2}=\mathrm{Pr}\left({\left|g_{kD2}\right|}^{2}>\frac{\zeta }{\rho_{R}}\right)=\exp\left(-\frac{\zeta }{\rho_{R}\lambda_{kD2}}.\right) \end{array}$$

It is constrained by $\alpha_{2}\rho_{E}{\left|h_{E}\right|}^{2}<\frac{\alpha_{2}}{\alpha_{1}}\to {\left|h_{E}\right|}^{2}<\frac{1}{\alpha_{1}\rho_{E}}$. In this situation, it can be rewritten as

(36) $$\begin{array}{cc} H_{1}\times H_{2} & =E_{{\left|h_{E}\right|}^{2}}\left\{\underset{H_{1}}{\underset{︸}{\exp\left(-\frac{\zeta }{\left(\alpha_{2}-\delta \alpha_{1}\right)\rho_{S}\lambda_{SR1}}\right)}}\times \underset{H_{2}}{\underset{︸}{\exp\left(-\frac{\zeta }{\rho_{R}\lambda_{kD2}}\right)}},\zeta <\frac{\alpha_{2}}{\alpha_{1}}\right\}\\ & =E_{{\left|h_{E}\right|}^{2}}\left\{\exp\left(-\frac{\alpha_{2}\rho_{E}{\left|h_{E}\right|}^{2}}{\left(\alpha_{2}-\alpha_{2}\rho_{E}{\left|h_{E}\right|}^{2}\alpha_{1}\right)\rho_{S}\lambda_{SR1}}\right)\times \exp\left(-\frac{\alpha_{2}\rho_{E}{\left|h_{E}\right|}^{2}}{\rho_{R}\lambda_{kD2}}\right),{\left|h_{E}\right|}^{2}<\frac{1}{\alpha_{1}\rho_{E}}\right\}\\ & =\frac{1}{\lambda_{E}}\int_{0}^{\frac{1}{\alpha_{1}\rho_{E}}}\exp\left(-\frac{\alpha_{2}\rho_{E}x}{\left(\alpha_{2}-\alpha_{1}\alpha_{2}\rho_{E}x\right)\rho_{S}\lambda_{SR1}}-\frac{\alpha_{2}\rho_{E}x}{\rho_{R}\lambda_{kD2}}-\frac{x}{\lambda_{E}}\right)dx. \end{array}$$

We formulate *H* as

(37) $$\begin{array}{c} H=1-\prod_{k=1}^{K}\left(1-\int_{0}^{\frac{1}{\alpha_{1}\rho_{E}}}\exp\left(-\frac{\alpha_{2}\rho_{E}x}{\left(\alpha_{2}-\alpha_{1}\alpha_{2}\rho_{E}x\right)\rho_{S}\lambda_{SR1}}-\frac{\alpha_{2}\rho_{E}x}{\rho_{R}\lambda_{kD2}}-\frac{x}{\lambda_{E}}\right)dx\right). \end{array}$$

Therefore, the SPSC evaluation at D2 can be determined by

(38) $$\begin{array}{cc} P_{SPSC2}^{NOMA} & =\frac{\rho_{R}\lambda_{D12}}{\left(\rho_{R}\lambda_{D12}+\alpha_{2}\rho_{E}\lambda_{E}\right)\alpha_{1}\rho_{E}\lambda_{E}}\exp\left(\frac{1}{\alpha_{1}\rho_{S}\lambda_{D1}}-\frac{1}{\alpha_{1}\rho_{E}\lambda_{E}}\right)q\left(v_{1}\right)\\ & \times \left(1-\prod_{k=1}^{K}\left(1-\int_{0}^{\frac{1}{\alpha_{1}\rho_{E}}}\exp\left(-\frac{\alpha_{2}\rho_{E}x}{\left(\alpha_{2}-\alpha_{1}\alpha_{2}\rho_{E}x\right)\rho_{S}\lambda_{SR1}}-\frac{\alpha_{2}\rho_{E}x}{\rho_{R}\lambda_{kD2}}-\frac{x}{\lambda_{E}}\right)dx\right)\right), \end{array}$$

where $q\left(v_{1}\right)=\int_{0}^{1}\exp\left(-\frac{1}{\alpha_{1}v_{1}\rho_{S}\lambda_{D1}}+\frac{v_{1}}{\alpha_{1}\rho_{E}\lambda_{E}}\right)dv_{1},\zeta =\alpha_{2}\rho_{E}{\left|h_{E}\right|}^{2}$.

## 5. Optimization and Studying OMA as Benchmark

### 5.1. Selection of $\alpha_{1}$ for NOMA Transmission

In this section, we perform a numerical search for the value of $\alpha_{1}$ that minimizes outage performance. However, these derived expressions of outage probability can not exhibit optimal $\alpha_{1}$. Fortunately, it can show an approximation to $\alpha_{1}$ obtained in a simple manner from the following observations

(39) $$\begin{array}{c} \gamma_{SD1,x1}^{NOMA}\geq \varepsilon_{2}\Rightarrow \vartheta_{SD1,x1}\geq \frac{\varepsilon_{2}}{\alpha_{1}}, \end{array}$$

where $\vartheta_{SD1,x1}=\rho_{S}{\left|h_{D1}\right|}^{2}$, and

(40) $$\begin{array}{c} \gamma_{SD1,x2}^{NOMA}\geq \varepsilon_{2}\Rightarrow \vartheta_{SD1,x2}\geq \frac{\varepsilon_{2}}{\alpha_{2}-\alpha_{1}\varepsilon_{2}}, \end{array}$$

where $\varepsilon_{2}=2^{2R_{2}}$.

Clearly, the value of $\alpha_{1}$ which minimizes outage performance is equivalent with evaluation of $\vartheta_{SD1,x1}$ and $\vartheta_{SD1,x2}$ as below

(41) $$\begin{array}{c} \vartheta_{SD1,x2}=\vartheta_{SD1,x1}\Rightarrow \alpha_{1}=\frac{1}{2+\varepsilon_{2}}. \end{array}$$

Although our derivation is clearly an approximation computation, its accuracy will be verified later in the numerical results section. It is interesting to see that considered outage value does not depend on the instantaneous channel values and it depends only on the target rates of the two users.

### 5.2. Asymptotic Analysis

We first consider asymptotic SOP for D1. To investigate the asymptotic secrecy performance, we also provide an asymptotic SOP analysis.

From (18), at high SNR $\rho_{E}$ the SOP performance of D1 based NOMA system can be asymptotically expressed as

(42) $$\begin{array}{c} P_{SOP1-asy}^{NOMA}\approx \mathrm{Pr}\left(\frac{1+\frac{a_{2}}{a_{1}}}{1+\alpha_{1}\rho_{E}{\left|h_{E}\right|}^{2}}<2^{2R_{1}}\right)\approx \mathrm{Pr}\left({\left|h_{E}\right|}^{2}>\frac{1+\frac{a_{2}}{a_{1}}-\varepsilon_{1}}{\varepsilon_{1}\alpha_{1}\rho_{E}}\right)\approx \exp\left(-\frac{1+\frac{a_{2}}{a_{1}}-\varepsilon_{1}}{\varepsilon_{1}\alpha_{1}\rho_{E}\lambda_{E}}\right), \end{array}$$

where $\varepsilon_{1}=2^{2R_{1}}$.

Then, we perform asymptotic derivation for SOP of D2. Similarly, from (19), the asymptotic expression for a D2 is given by

(43) $$\begin{array}{c} P_{SOP2}^{NOMA}\approx \underset{U_{1}}{\underset{︸}{\mathrm{Pr}\left(\frac{1+\frac{a_{2}}{a_{1}}}{1+\gamma_{SE2}^{NOMA}}<2^{2R_{2}}\right)}}\underset{U_{2}}{\underset{︸}{\mathrm{Pr}\left(\frac{1+\frac{a_{2}}{a_{1}}}{1+\gamma_{SE2*}^{NOMA}}\geq 2^{2R_{2}}\right)}}. \end{array}$$

From (43), we can calculate $U_{1}$ as

(44) $$\begin{array}{c} U_{1}=\mathrm{Pr}\left(\frac{1+\frac{a_{2}}{a_{1}}}{1+\alpha_{2}\rho_{E}{\left|h_{E}\right|}^{2}}<2^{2R_{2}}\right)\approx \exp\left(-\frac{1+\frac{a_{2}}{a_{1}}-\varepsilon_{2}}{\varepsilon_{2}\alpha_{2}\rho_{E}\lambda_{E}}\right). \end{array}$$

Similarly, with $U_{2}$ we get

(45) $$\begin{array}{cc} U_{2} & =\mathrm{Pr}\left(\frac{1+\frac{a_{2}}{a_{1}}}{1+\gamma_{SE2*}^{NOMA}}<2^{2R_{2}}\right)=\prod_{k=1}^{K}\mathrm{Pr}\left({\left|h_{E}\right|}^{2}>\frac{1+\frac{a_{2}}{a_{1}}-\varepsilon_{2}}{\varepsilon_{2}\alpha_{2}\rho_{E}}\right)\\ & =\prod_{k=1}^{K}\left(\exp\left(-\frac{1+\frac{a_{2}}{a_{1}}-\varepsilon_{2}}{\varepsilon_{2}\alpha_{2}\rho_{E}\lambda_{E}}\right)\right). \end{array}$$

Replacing (44) and (45) into (43) leads to

(46) $$\begin{array}{c} P_{SOP2}^{NOMA}\approx \exp\left(-\frac{1+\frac{a_{2}}{a_{1}}-\varepsilon_{2}}{\varepsilon_{2}\alpha_{2}\rho_{E}\lambda_{E}}\right)\prod_{k=1}^{K}\left(\exp\left(-\frac{1+\frac{a_{2}}{a_{1}}-\varepsilon_{2}}{\varepsilon_{2}\alpha_{2}\rho_{E}\lambda_{E}}\right)\right). \end{array}$$

### 5.3. Consideration on OMA as Benchmark

As a traditional multiple access scheme, OMA is still deployed in a huge number of applications. It is further considered an advantage of NOMA compared with older counterpart, i.e., OMA. Although security concerns in OMA scheme are studied in the literature, this paper carefully presents the main computations to make such comparisons clearer. In OMA, we first compute SNR to detect $x_{1}$ from the BS to D1 as

(47) $$\begin{array}{c} \gamma_{SD1,x1}^{OMA}=\frac{P_{S}{\left|h_{D1}\right|}^{2}}{\sigma_{0}^{2}}=\rho_{S}{\left|h_{D1}\right|}^{2}. \end{array}$$

To detect $x_{2}$ from the BS to relay, it is required to calculate SNR as

(48) $$\begin{array}{c} \gamma_{SR,x2}^{OMA}=\frac{P_{S}{\left|h_{SR1}\right|}^{2}}{\sigma_{0}^{2}}=\rho_{S}{\left|h_{SRk}\right|}^{2}. \end{array}$$

We compute SNR to detect $x_{2}$ in second hop from relay to D2 as

(49) $$\begin{array}{c} \gamma_{RD2,x2}^{OMA}=\frac{P_{R}{\left|g_{kD2}\right|}^{2}}{\sigma_{0}^{2}}=\rho_{R}{\left|g_{kD2}\right|}^{2}. \end{array}$$

It is shown SNR to detect signal $x_{1}$, $x_{2}$ at E respectively

(50) $$\begin{array}{c} \gamma_{SE1}^{OMA}=\frac{P_{S}{\left|h_{E}\right|}^{2}}{\sigma_{E}^{2}}=\rho_{E}{\left|h_{E}\right|}^{2}, \end{array}$$

and

(51) $$\begin{array}{c} \gamma_{SE2}^{OMA}=\frac{P_{S}{\left|h_{E}\right|}^{2}}{\sigma_{E}^{2}}=\rho_{E}{\left|h_{E}\right|}^{2}. \end{array}$$

Similarly, the secrecy capacity for D1 in OMA is obtained as

(52) $$\begin{array}{c} C_{x1}^{OMA}={\left[\frac{1}{2}\log_{2}\left(\frac{1+\gamma_{SD1,x1}^{OMA}}{1+\gamma_{SE1}^{OMA}}\right)\right]}^{+} \end{array}.$$

The secrecy capacity for D2 in OMA is obtained as

(53) $$\begin{array}{c} C_{x*2}^{OMA}={\left[\frac{1}{4}\log_{2}\left(\frac{1+\gamma_{k*}^{OMA}}{1+\gamma_{SE2}^{OMA}}\right)\right]}^{+} \end{array}.$$

It is worth noting that the best relay node is selected by the following criterion $\gamma_{k*}^{OMA}=\max_{k=1,\ldots ,K}\left(\gamma_{k}^{OMA}\right)$ with $\gamma_{k}^{OMA}=\min\left(\gamma_{SR,x2}^{OMA},\gamma_{RD2,x2}^{OMA}\right)$.

In similar way, SOP at D1 in OMA scheme is given by

(54) $$\begin{array}{cc} P_{SOP1}^{OMA} & =1-\mathrm{Pr}\left(C_{x1}^{OMA}\geq \xi_{1}\right)=1-\mathrm{Pr}\left(\frac{1+\gamma_{SD1,x1}^{OMA}}{1+\gamma_{SE1}^{OMA}}\geq \xi_{1}\right)\\ & =1-\mathrm{Pr}\left({\left|h_{D1}\right|}^{2}\geq \frac{\xi_{1}\rho_{E}}{\rho_{S}}{\left|h_{E}\right|}^{2}+\frac{\xi_{1}-1}{\rho_{S}}\right). \end{array}$$

In next step, it is calculated as

(55) $$\begin{array}{cc} P_{SOP1}^{OMA} & =1-\mathrm{Pr}\left({\left|h_{D1}\right|}^{2}\geq A_{1}^{OMA}{\left|h_{E}\right|}^{2}+B_{1}^{OMA}\right)\\ & =1-\int_{0}^{\infty }\exp\left(-\frac{A_{1}^{OMA}x+B_{1}^{OMA}}{\lambda_{D1}}\right)\frac{1}{\lambda_{E}}\exp\left(-\frac{x}{\lambda_{E}}\right)dx\\ & =1-\frac{1}{\lambda_{E}}\exp\left(-\frac{B_{1}^{OMA}}{\lambda_{D1}}\right)\int_{0}^{\infty }\exp\left(-\left(\frac{A_{1}^{OMA}}{\lambda_{D1}}+\frac{1}{\lambda_{E}}\right)x\right)dx. \end{array}$$

Finally, SOP at D1 in this situation is given by

(56) $$\begin{array}{c} P_{SOP1}^{OMA}=1-\frac{\lambda_{D1}}{\lambda_{E}A_{1}^{OMA}+\lambda_{D1}}\exp\left(-\frac{B_{1}^{OMA}}{\lambda_{D1}}\right) \end{array},$$

where $\xi_{1}=2^{2R_{1}}$, $A_{1}^{OMA}=\frac{\xi_{1}\rho_{E}}{\rho_{S}},B_{1}^{OMA}=\frac{\xi_{1}-1}{\rho_{S}}$.

The SOP at D2 in OMA scheme is further computed as

(57) $$\begin{array}{cc} P_{SOP2}^{OMA} & =\mathrm{Pr}\left(C_{x*2}^{OMA}<R_{2}\right)=\mathrm{Pr}\left(\frac{1+\gamma_{k*}^{OMA}}{1+\gamma_{SE2}^{OMA}}<2^{4R_{2}}\right)\\ & =\mathrm{Pr}\left(\underset{\nu^{*}}{\underset{︸}{\max_{k=1,\ldots ,K}\left(\min\left(\rho_{S}{\left|h_{SR1}\right|}^{2},\rho_{R}{\left|g_{kD2}\right|}^{2}\right)\right)}}<\psi {\left|h_{E}\right|}^{2}+\mu \right). \end{array}$$

And then, it is rewritten as

(58) $$\begin{array}{cc} P_{SOP2}^{OMA} & =\int_{0}^{\infty }\left(1-F_{\nu^{*}}\left(-\eta y_{1}\right)\right)f_{{\left|h_{E}\right|}^{2}}\left(x\right)dx\\ & =\int_{0}^{\infty }\left[1-{\left(1-\exp\left(-\eta \left(\psi x+\mu \right)\right)\right)}^{k}\right]\frac{1}{\lambda_{E}}\exp\left(\frac{-x}{\lambda_{E}}\right)dx\\ & =\frac{1}{\lambda_{E}}\exp\left(-k\eta \mu \right)\\ & \times \int_{0}^{\infty }\left(\sum_{k=1}^{K}\left(\begin{array}{c} K\\ k \end{array}\right){\left(-1\right)}^{k-1}\exp\left(-\left(k\eta \psi +\frac{1}{\lambda_{E}}\right)x\right)\right)dx\\ & =1-\exp\left(-k\eta \mu \right)\sum_{k=1}^{K}\left(\begin{array}{c} K\\ k \end{array}\right){\left(-1\right)}^{k-1}\frac{1}{k\eta \psi \lambda_{E}+1}, \end{array}$$

where $\psi =2^{4R_{2}}\rho_{E},\mu =2^{4R_{2}}-1,\eta =\frac{1}{\rho_{S}\lambda_{SRk}}+\frac{1}{\rho_{R}\lambda_{kD2}}$.

The SOP for secure performance evaluation of whole OMA is given as

(59) $$\begin{array}{c} OP_{OMA}=1-\left(1-OP_{1-OMA}\right)\left(1-OP_{2-OMA}\right). \end{array}$$

Regarding SPSC analysis for an OMA scenario, we have the following equation in similar computation. We first present SPSC metric at D1 as

(60) $$\begin{array}{cc} P_{SPSC1}^{OMA} & =\mathrm{Pr}\left(C_{x1}^{OMA}>0\right)=\mathrm{Pr}\left(\gamma_{SD1,x1}^{OMA}>\gamma_{SE1}^{OMA}\right)\\ & =\frac{1}{\lambda_{E}}\int_{0}^{\infty }\exp\left(-\left(\frac{\rho_{E}}{\rho_{S}\lambda_{D1}}+\frac{1}{\lambda_{E}}\right)x\right)dx=\frac{\rho_{S}\lambda_{D1}}{\rho_{E}\lambda_{E}+\rho_{S}\lambda_{D1}}. \end{array}$$

Furthermore, the expression of SPSC metric can be derived at D2 as

(61) $$\begin{array}{cc} P_{SPSC2}^{OMA} & =\int_{0}^{\infty }\left(1-F_{\nu^{*}}\left(-\eta y_{2}\right)\right)f_{{\left|h_{E}\right|}^{2}}\left(x\right)dx\\ & =\int_{0}^{\infty }\left[1-{\left(1-\exp\left(-\eta \rho_{E}x\right)\right)}^{k}\right]\frac{1}{\lambda_{E}}\exp\left(\frac{-x}{\lambda_{E}}\right)dx\\ & =\frac{1}{\lambda_{E}}\int_{0}^{\infty }\left(\sum_{k=1}^{K}\left(\begin{array}{c} K\\ k \end{array}\right){\left(-1\right)}^{k-1}\exp\left(-\left(k\eta \rho_{E}+\frac{1}{\lambda_{E}}\right)x\right)\right)dx\\ & =1-\sum_{k=1}^{K}\left(\begin{array}{c} K\\ k \end{array}\right){\left(-1\right)}^{k-1}\frac{1}{k\eta \rho_{E}\lambda_{E}+1}, \end{array}$$

where $\eta =\frac{1}{\rho_{S}\lambda_{SRk}}+\frac{1}{\rho_{R}\lambda_{kD2}}$.

## 6. Numerical Results

In this section, we provide numerical examples to evaluate the secrecy performance of RS-NOMA under impact of eavesdropper based on two system metrics including SOP and SPSC. Specifically, we investigate these metrics by considering the effects of transmit SNR, fixed power allocation factors, the number of relays, channel gains.

**Figure 2:**
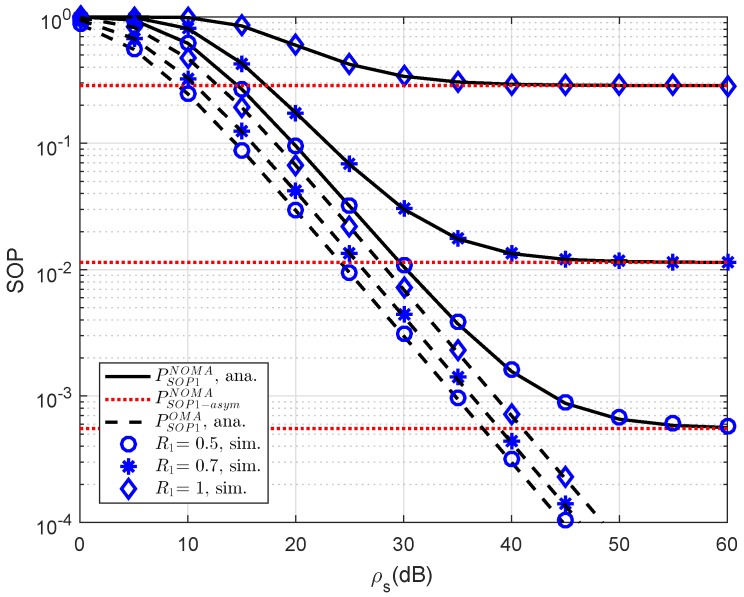
Comparison study on SOP of NOMA and OMA for User D1 versus $\rho_{S}=\rho_{R}$ as changing $R_{1}$ (${\lambda_{D}}_{1}=\lambda_{E}=1$, $\rho_{E}=0$ dB, $R_{2}=1$).

As an important parameter of NOMA, the impact of different threshold rates on the SOP performance of user D1 is simulated in Figure 2. The reason for such observation is that the threshold rate is the limited secure capacity as performing probability calculation. At high threshold rate, the performance gap between NOMA and OMA can be observed clearly. In addition, asymptotic evaluation shows that outage behavior is constant because such outage does not depend on $\rho_{S}$. This observation can be seen in the following experiments.

**Figure 3:**
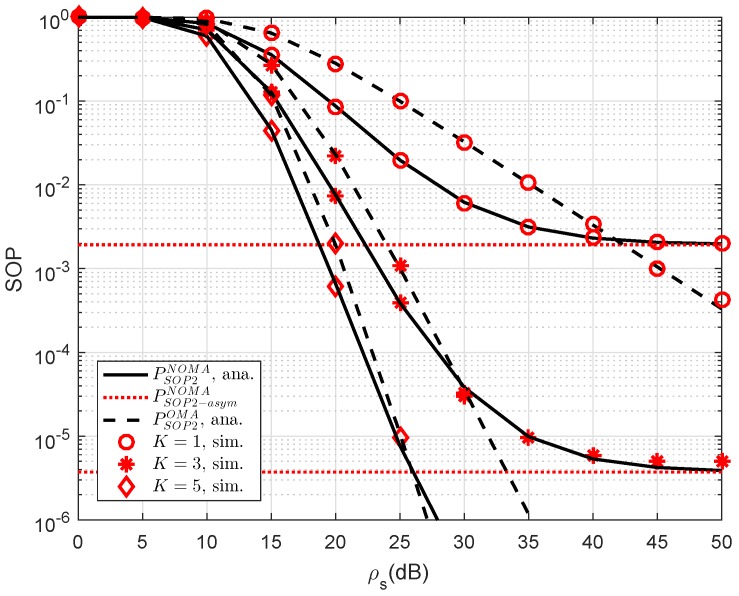
Comparison study on SOP of NOMA and OMA for User D2 versus $\rho_{S}=\rho_{R}$ as changing *K* (${\lambda_{D}}_{1}={\lambda_{D}}_{12}=\lambda_{SRk}={\lambda_{kD}}_{2}=\lambda_{E}=1$, $\rho_{E}=-10$ dB, $R_{2}=1$).

Another observation is that the impact of the number of relays selected to forward signal to user D2. As a further development, Figure 3 plots the SOP of NOMA scheme versus a different number of relays. As observed from the figure, we can see that the higher number of selected relays also strongly affect secure performance of RS-NOMA scheme compared with small variations at OMA. The most important thing is that the RS-NOMA furnishes with K=5 relay providing remarkable improvement in secure outage performance. This is due to the fact that there are more chances to achieve improved signal to serve far NOMA user. This observation confirms a role of relay selection to enhanced secure performance in the considered RS-NOMA.

**Figure 4:**
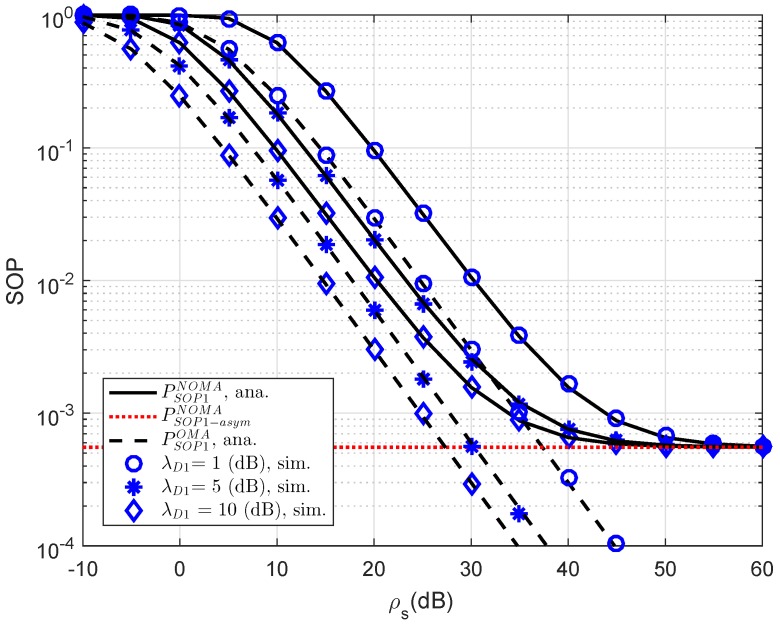
Comparison study on SOP of NOMA and OMA for User D1 versus transmit $\rho_{S}=\rho_{R}$ as varying $\lambda_{D1}$.

Figure 4 plots the outage probability of RS-NOMA and OMA schemes versus SNR for simulation settings with $\lambda_{E}=1$, $\rho_{E}=0$ dB, $R_{1}=0.5$, $R_{2}=1$. Obviously, the outage probability curves match precisely with the Monte Carlo simulation results. In this observation, the performance gap between NOMA and OMA is small as changing channel gain of link S-D1. This is in contrast with Figure 4, which shows larger a performance gap between NOMA and OMA for secure consideration at D2.

**Figure 5:**
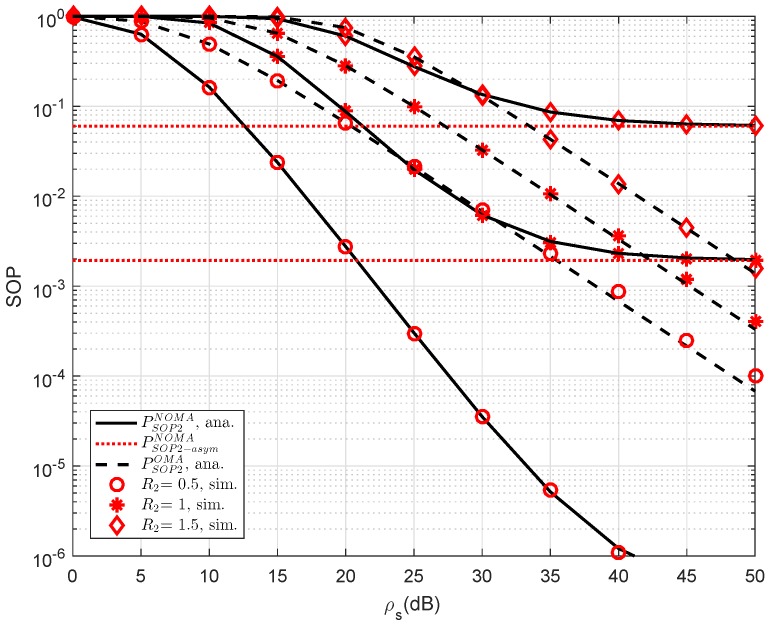
SOP of NOMA and OMA for User D2 versus $\rho_{S}=\rho_{R}$ as varying $R_{2}$.

In Figure 5, the SOP performance of the RS-NOMA and OMA schemes with different threshold rates at D2 are compared to provide an impact of the required rates on secure performance. We setup the main parameters as ${\lambda_{D}}_{1}={\lambda_{D}}_{12}=\lambda_{SRk}={\lambda_{kD}}_{2}=\lambda_{E}=1$, $\rho_{E}=-10$ dB, K=1. It can be seen from both figures that the proposed RS-NOMA scheme can remarkably enhance the secure performance compared to the OMA scheme. Performance gaps between NOMA and OMA can be seen clearly at higher threshold rate $R_{2}$.

**Figure 6:**
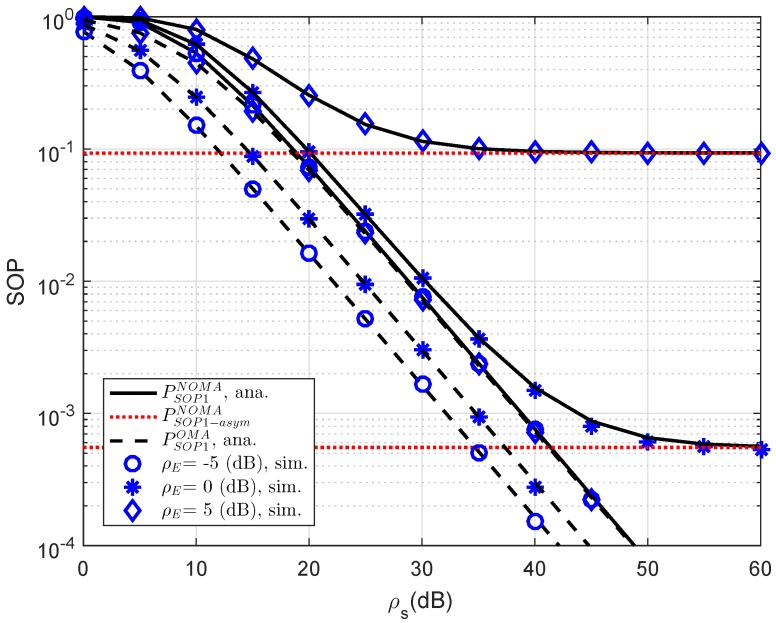
Comparison study of SOP for NOMA and OMA for User D1 versus $\rho_{S}=\rho_{R}$ as varying $\rho_{E}$.

**Figure 7:**
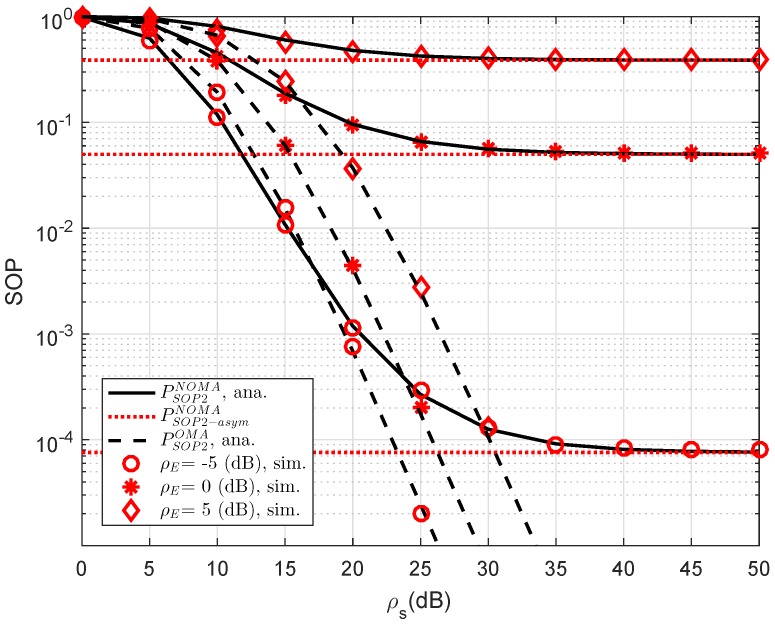
Comparison study of SOP for NOMA and OMA for User D2 versus $\rho_{S}=\rho_{R}$ as varying $\rho_{E}$.

**Figure 8:**
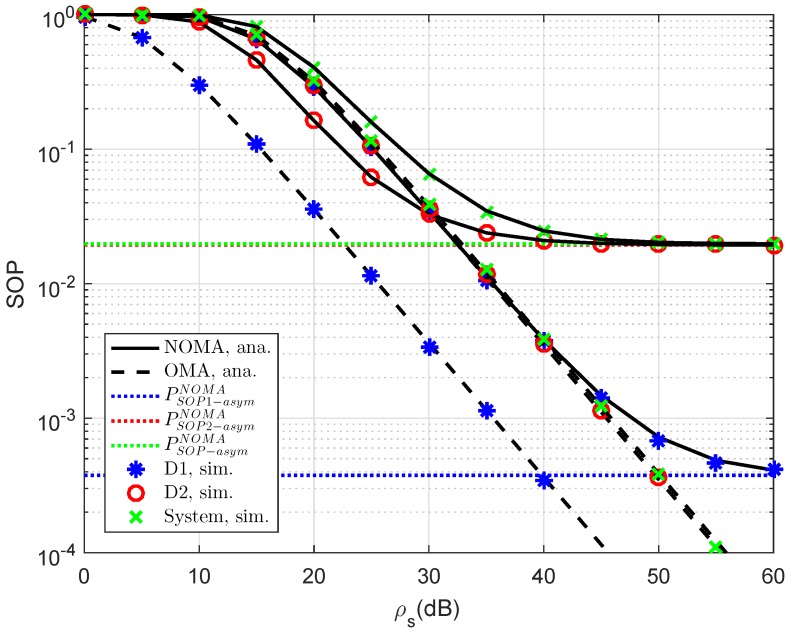
Comparison study of SOP in several cases versus $\rho_{S}=\rho_{R}$ (${\lambda_{D}}_{1}={\lambda_{D}}_{12}=\lambda_{SRk}={\lambda_{kD}}_{2}=\lambda_{E}=1$, $\rho_{E}=-8$ dB, K=1, $R_{1}=R_{2}=1$).

In Figure 6, we compare the secure performance for the RS-NOMA and OMA schemes with different strong levels of eavesdroppers. To perform the simulation, the required parameters are summarized as ${\lambda_{D}}_{1}=\lambda_{E}=1$, $R_{1}=0.5$, $R_{2}=1$. It can be evidently seen that SOP in the OMA is better than that in the RS-NOMA scheme. The main reason for this is that the cooperative NOMA network is sensitive to the relation between the target data rates and power allocation. In a similar trend, we see the performance gap at user D2 as in Figure 7. In this situation, the simulated parameters are shown in this case as ${\lambda_{D}}_{1}={\lambda_{D}}_{12}=\lambda_{SRk}={\lambda_{kD}}_{2}=\lambda_{E}=1$, $R_{2}=0.5$, K=3. To provide more insights, the secure performance of the whole system needs be considered. In Figure 8, the curves of SOP are illustrated to show performance gaps among these cases including User D1, User D2 and the whole NOMA system.

**Figure 9:**
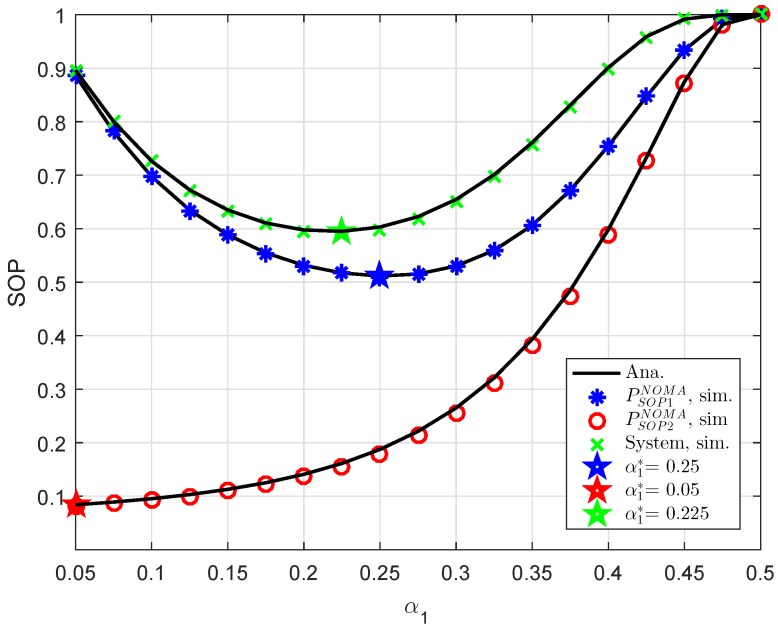
Optimal SOP in several cases with indication of optimal value regarding $\alpha_{1}$ (${\lambda_{D}}_{1}={\lambda_{D}}_{12}=\lambda_{SRk}={\lambda_{kD}}_{2}=\lambda_{E}=1$, $\rho_{E}=-5$ dB, K=1, $R_{1}=R_{2}=0.5$).

In Figure 9, an optimal value of power allocation factor, i.e., $\alpha_{1}$ can be checked by a numerical method. It can be confirmed that our derivation in an approximate manner is similar to numerical value obtained. This is the guideline for designing NOMA to achieve the lowest outage performance.

**Figure 10:**
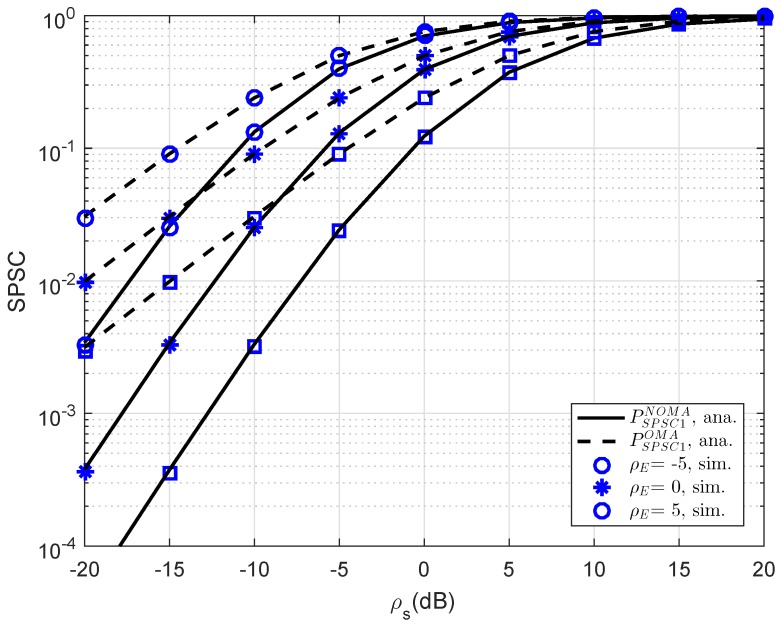
Comparison study of SPSC in several cases versus $\rho_{S}=\rho_{R}$ at D1 as setting different values of $\rho_{E}$ (${\lambda_{D}}_{1}=\lambda_{E}=1$, $R_{1}=0.5$, $R_{2}=1$).

In Figure 10, further simulation is performed for consideration at D1; the SPSC performance versus transmit SNR is presented. As can be seen, at lower SNR regime, SPSC performance between OMA and NOMA is similar. This observation will change at higher SNR. The strong characterization of eavesdropper leads to varying SPSC performance. As seen in other simulations, this result verifies the exactness of the analytical computations presented in the previous section.

**Figure 11:**
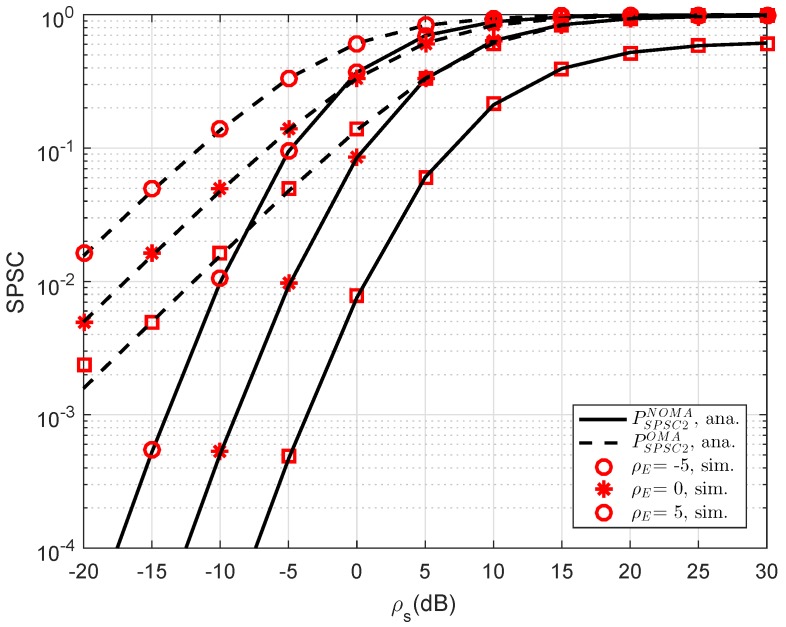
SPSC performance in several cases versus $\rho_{S}=\rho_{R}$ as different choices of $\rho_{E}$ (${\lambda_{D}}_{1}={\lambda_{D}}_{12}=\lambda_{SRk}={\lambda_{kD}}_{2}=\lambda_{E}=1$, K=1, $R_{1}=0.5$,$R_{2}=1$).

In Figure 11, the curves of SPSC versus transmit SNR at D2 are presented. As can be seen, the analytical results can match the simulations very well. Obviously, by varying channel gains of the eavesdropper, the SPSC will be changed. Meanwhile, the performance gap between OMA and NOMA in such SPSC is linear in the range of SNR from −20 dB to 5 dB and it does not exist if the SNR is greater than 10 dB. Like previous simulations, this result coincides with the analysis in analytical computations presented in the previous section.

## 7. Conclusions

In this study, the closed-form expressions are derived in a scenario of relaying network deploying NOMA. In such NOMA, relay in group is selected to evaluate secure performance in situations regarding the existence of secrecy probability in such RS-NOMA. In this scenario, we considered a system with an eavesdropper, multiple-relay, two NOMA users, and a base station. As an important achievement, the best relay selection criteria was recommended to enhance system secrecy performance against eavesdropping attacks. By evaluating the effects of various indicators of the system, we investigated two main metrics, the SPSC and the SOP and then secrecy performance analysis is achieved. In addition, we further demonstrated the accuracy of the analysis using Monte Carlo simulations. In addition, we confirmed the advantage of NOMA scheme compared with OMA at specific values of simulated parameters. For future work, multiple antenna at the base station and multiple eavesdroppers should be examined together with relaying techniques to illustrate a practical implementation of RS-NOMA.

## Appendix A

**Proof of Proposition** **A1.** We first compute SOP based on the concerned definition as

(A1) $$\begin{array}{cc} P_{SOP1}^{NOMA} & =1-\mathrm{Pr}\left(C_{x1}^{NOMA}\geq R_{1}\right)\\ & =1-\mathrm{Pr}\left(\frac{1}{2}\log_{2}\left(min\left(\frac{1+\gamma_{SD1,x1}^{NOMA}}{1+\gamma_{SE1}^{NOMA}},\frac{1+\gamma_{SD1,x2}^{NOMA}}{1+\gamma_{SE1}^{NOMA}}\right)\right)\geq R_{1}\right)\\ & \approx 1-\underset{X_{1}}{\underset{︸}{\mathrm{Pr}\left(\gamma_{SD1,x1}^{NOMA}\geq 2^{2R_{1}}\left(1+\gamma_{SE1}^{NOMA}\right)-1\right)}}\underset{X_{2}}{\underset{︸}{\mathrm{Pr}\left(\gamma_{SD1,x2}^{NOMA}\geq 2^{2R_{1}}\left(1+\gamma_{SE1}^{NOMA}\right)-1\right)}}. \end{array}$$

To compute such outage, $X_{1}$ can be first calculated as

(A2) $$\begin{array}{cc} X_{1} & =\mathrm{Pr}\left({\left|h_{D1}\right|}^{2}\geq \frac{\varphi_{1}{\left|h_{E}\right|}^{2}+\psi_{1}}{\alpha_{1}\rho_{S}}\right)\\ & =\int_{0}^{\infty }\exp\left(-\frac{\varphi_{1}{\left|h_{E}\right|}^{2}+\psi_{1}}{\alpha_{1}\rho_{S}\lambda_{D1}}\right)\frac{1}{\lambda_{E}}\exp\left(-\frac{x}{\lambda_{E}}\right)dx\\ & =\frac{\alpha_{1}\rho_{S}\lambda_{D1}}{\alpha_{1}\rho_{S}\lambda_{D1}+\varepsilon_{1}\lambda_{E}}\exp\left(-\frac{\psi_{1}}{\alpha_{1}\rho_{S}\lambda_{D1}}\right). \end{array}$$

where $\varphi_{1}=2^{2R_{1}}\alpha_{1}\rho_{E},\psi_{1}=2^{2R_{1}}-1$. In addition, $X_{2}$ can be expressed by

(A3) $$\begin{array}{c} X_{2}=\mathrm{Pr}\left({\left|h_{D1}\right|}^{2}\geq \frac{\varphi_{1}{\left|h_{E}\right|}^{2}+\psi_{1}}{\rho_{S}\left(\alpha_{2}-\psi_{1}\alpha_{1}-\varphi_{1}\alpha_{1}{\left|h_{E}\right|}^{2}\right)}\right). \end{array}$$

It is noted that strict constraint here is $\rho_{S}\left(\alpha_{2}-\psi_{1}\alpha_{1}-\varphi_{1}\alpha_{1}{\left|h_{E}\right|}^{2}\right)>0\to {\left|h_{E}\right|}^{2}<\frac{\alpha_{2}-\psi_{1}\alpha_{1}}{\varphi_{1}\alpha_{1}}$ then $X_{2}$ can be further computed by

(A4) $$\begin{array}{c} X_{2}=\int_{0}^{\frac{\alpha_{2}-\psi_{1}\alpha_{1}}{\varphi_{1}\alpha_{1}}}\exp\left(-\frac{\varphi_{1}x+\psi_{1}}{\left(\alpha_{2}-\psi_{1}\alpha_{1}-\varphi_{1}\alpha_{1}x\right)\rho_{S}\lambda_{D1}}\right)\frac{1}{\lambda_{E}}\exp\left(-\frac{x}{\lambda_{E}}\right)dx. \end{array}$$

Next, new variable can be seen as $t_{1}=\alpha_{2}-\psi_{1}\alpha_{1}-\varphi_{1}\alpha_{1}x\to x=\frac{\alpha_{2}-\psi_{1}\alpha_{1}-t_{1}}{\varphi_{1}\alpha_{1}}$ then $X_{2}$ can be re-expressed by

(A5) $$\begin{array}{cc} X_{2} & =\frac{1}{\varphi_{1}\alpha_{1}\lambda_{E}}\int_{0}^{\alpha_{2}-\psi_{1}\alpha_{1}}\exp\left(-\frac{\alpha_{2}-t_{1}}{\alpha_{1}t_{1}\rho_{S}\lambda_{D1}}\right)\exp\left(-\frac{\alpha_{2}-\psi_{1}\alpha_{1}-t_{1}}{\varphi_{1}\alpha_{1}\lambda_{E}}\right)dt_{1}\\ & =\frac{1}{\varphi_{1}\alpha_{1}\lambda_{E}}\exp\left(\frac{1}{\alpha_{1}\rho_{S}\lambda_{D1}}-\frac{\alpha_{2}-\psi_{1}\alpha_{1}}{\varphi_{1}\alpha_{1}\lambda_{E}}\right)U\left(t_{1}\right). \end{array}$$

After performing simple manipulations, it can be obtained that

(A6) $$\begin{array}{c} P_{SOP1}^{NOMA}=1-\frac{\alpha_{1}\rho_{S}\lambda_{D1}}{\left(\alpha_{1}\rho_{S}\lambda_{D1}+\varphi_{1}\lambda_{E}\right)\varphi_{1}\alpha_{1}\lambda_{E}}\exp\left(-\frac{\psi_{1}}{\alpha_{1}\rho_{S}\lambda_{D1}}+\frac{1}{\alpha_{1}\rho_{S}\lambda_{D1}}-\frac{\alpha_{2}-\psi_{1}\alpha_{1}}{\varphi_{1}\alpha_{1}\lambda_{E}}\right)U\left(t_{1}\right). \end{array}$$

This is end of the proof. ☐

## Appendix B

**Proof of Proposition** **A2.** From the definition, it can be expressed SOP as

(A7) $$\begin{array}{cc} P_{SOP2}^{NOMA} & =\mathrm{Pr}\left(C_{x2}^{NOMA}<R_{2}\right)\\ & =\mathrm{Pr}\left(\gamma_{SD1,x2}^{NOMA}<2^{2R_{2}}\left(1+\gamma_{SE2}^{NOMA}\right)-1\cup \gamma_{D12,x2}^{NOMA}<2^{2R_{2}}\left(1+\gamma_{SE2}^{NOMA}\right)-1\right)\\ & \times \mathrm{Pr}\left(\gamma_{SRk*,x2}^{NOMA}<2^{2R_{2}}\left(1+\gamma_{SE2}^{NOMA}\right)-1\cup \gamma_{Rk*D2,x2}^{NOMA}<2^{2R_{2}}\left(1+\gamma_{SE2}^{NOMA}\right)-1\right)\\ & =\underset{Q_{1}}{\underset{︸}{\left(1-\mathrm{Pr}\left(\gamma_{SD1,x2}^{NOMA}\geq 2^{2R_{2}}\left(1+\gamma_{SE2}^{NOMA}\right)-1,\gamma_{D12,x2}^{NOMA}\geq 2^{2R_{2}}\left(1+\gamma_{SE2}^{NOMA}\right)-1\right)\right)}}\\ & \times \underset{Q_{2}}{\underset{︸}{\prod_{k=1}^{K}\left(1-\mathrm{Pr}\left(\gamma_{SRk,x2}^{NOMA}\geq 2^{2R_{2}}\left(1+\gamma_{SE2}^{NOMA}\right)-1,\gamma_{RkD2,x2}^{NOMA}\geq 2^{2R_{2}}\left(1+\gamma_{SE2}^{NOMA}\right)-1\right)\right)}}. \end{array}$$

In this situation, $R_{1}$ can be written as

(A8) $$\begin{array}{cc} Q_{1} & =1-\mathrm{Pr}\left(\gamma_{SD1,x2}^{NOMA}\geq 2^{2R_{2}}\left(1+\gamma_{SE2}^{NOMA}\right)-1,\gamma_{D12,x2}^{NOMA}\geq 2^{2R_{2}}\left(1+\gamma_{SE2}^{NOMA}\right)-1\right)\\ & =1-\underset{J_{1}}{\underset{︸}{\mathrm{Pr}\left({\left|h_{D1}\right|}^{2}\geq \frac{\varphi_{2}{\left|h_{E}\right|}^{2}+\psi_{2}}{\alpha_{2}\rho_{S}-\psi_{2}\alpha_{1}\rho_{S}-\varphi_{2}\alpha_{1}\rho_{S}{\left|h_{E}\right|}^{2}}\right)}}\underset{J_{2}}{\underset{︸}{\mathrm{Pr}\left({\left|g_{D12}\right|}^{2}\geq \frac{\varphi_{2}{\left|h_{E}\right|}^{2}+\psi_{2}}{\rho_{R}}\right)}}. \end{array}$$

This case requires the constraint as $\alpha_{2}\rho_{S}-\psi_{2}\alpha_{1}\rho_{S}-\varphi_{2}\alpha_{1}\rho_{S}{\left|h_{E}\right|}^{2}>0\to {\left|h_{E}\right|}^{2}<\frac{\alpha_{2}\rho_{S}-\psi_{2}\alpha_{1}\rho_{S}}{\varphi_{2}\alpha_{1}\rho_{S}}$ then $J_{1}$ can be expressed by

(A9) $$\begin{array}{c} J_{1}=\frac{1}{\lambda_{E}}\int_{0}^{\frac{\alpha_{2}\rho_{S}-\psi_{2}\alpha_{1}\rho_{S}}{\varepsilon_{2}\alpha_{1}\rho_{S}}}\exp\left(-\frac{\varphi_{2}x+\psi_{2}}{\left(\alpha_{2}\rho_{S}-\psi_{2}\alpha_{1}\rho_{S}-\varphi_{2}\alpha_{1}\rho_{S}x\right)\lambda_{D1}}-\frac{x}{\lambda_{E}}\right)dx. \end{array}$$

To further computation, we set new variable as $t_{2}=\alpha_{2}\rho_{S}-\psi_{2}\alpha_{1}\rho_{S}-\varphi_{2}\alpha_{1}\rho_{S}x\to x=\frac{\alpha_{2}\rho_{S}-\psi_{2}\alpha_{1}\rho_{S}-t_{2}}{\varphi_{2}\alpha_{1}\rho_{S}}$ then it can be expressed by

(A10) $$\begin{array}{c} J_{1}=\frac{1}{\varphi_{2}\alpha_{1}\rho_{S}\lambda_{E}}\exp\left(\frac{1}{\alpha_{1}\rho_{S}\lambda_{D1}}-\frac{\alpha_{2}\rho_{S}-\psi_{2}\alpha_{1}\rho_{S}}{\varphi_{2}\alpha_{1}\rho_{S}\lambda_{E}}\right)q\left(t_{2}\right). \end{array}$$

In this step, it can be shown $J_{2}$ as

(A11) $$\begin{array}{cc} J_{2} & =\mathrm{Pr}\left({\left|h_{D12}\right|}^{2}\geq \frac{\varphi_{2}{\left|h_{E}\right|}^{2}+\psi_{2}}{\rho_{R}}\right)\\ & =\int_{0}^{\infty }\exp\left(-\frac{\varphi_{2}x+\psi_{2}}{\rho_{R}\lambda_{D12}}\right)\frac{1}{\lambda_{E}}\exp\left(-\frac{x}{\lambda_{E}}\right)dx\\ & =\frac{\rho_{R}\lambda_{D12}}{\rho_{R}\lambda_{D12}+\varphi_{2}\lambda_{E}}\exp\left(-\frac{\psi_{2}}{\rho_{R}\lambda_{D12}}\right). \end{array}$$

From (A10) and (A11), it can be obtained $R_{1}$ as

(A12) $$\begin{array}{c} Q_{1}=1-\frac{\rho_{R}\lambda_{D12}}{\left(\rho_{R}\lambda_{D12}+\varphi_{2}\lambda_{E}\right)\varphi_{2}\alpha_{1}\rho_{S}\lambda_{E}}\exp\left(\frac{1}{\alpha_{1}\rho_{S}\lambda_{D1}}-\frac{\alpha_{2}\rho_{S}-\psi_{2}\alpha_{1}\rho_{S}}{\varphi_{2}\alpha_{1}\rho_{S}\lambda_{E}}-\frac{\psi_{2}}{\rho_{R}\lambda_{D12}}\right)q\left(t_{2}\right), \end{array}$$

where $\varphi_{2}=2^{2R_{2}}\alpha_{2}\rho_{E},\psi_{2}=2^{2R_{2}}-1,q\left(t_{2}\right)=\int_{0}^{\alpha_{2}\rho_{S}-\psi_{2}\alpha_{1}\rho_{S}}\exp\left(-\frac{\alpha_{2}\rho_{S}}{\alpha_{1}\rho_{S}t_{2}\lambda_{D1}}+\frac{t_{2}}{\varphi_{2}\alpha_{1}\rho_{S}\lambda_{E}}\right)dt_{2}$. Then, $R_{2}$ can be calculated as

(A13) $$\begin{array}{c} Q_{2}=\prod_{k=1}^{K}\left(1-\underset{Y_{1}}{\underset{︸}{\mathrm{Pr}\left({\left|h_{SR1}\right|}^{2}\geq \frac{\varphi_{2}{\left|h_{E}\right|}^{2}+\psi_{2}}{\alpha_{2}\rho_{S}-\psi_{2}\alpha_{1}\rho_{S}-\varphi_{2}\alpha_{1}\rho_{S}{\left|h_{E}\right|}^{2}}\right)}}\underset{Y_{2}}{\underset{︸}{\mathrm{Pr}\left({\left|g_{kD2}\right|}^{2}\geq \frac{\varphi_{2}{\left|h_{E}\right|}^{2}+\psi_{2}}{\rho_{R}}\right)}}\right). \end{array}$$

Such outage event need the condition as $\alpha_{2}\rho_{S}-\psi_{2}\alpha_{1}\rho_{S}-\varphi_{2}\alpha_{1}\rho_{S}{\left|h_{E}\right|}^{2}>0\to {\left|h_{E}\right|}^{2}<\frac{\alpha_{2}-\psi_{2}\alpha_{1}}{\varphi_{2}\alpha_{1}}$ then $Y_{1}$ can be expressed by

(A14) $$\begin{array}{c} Y_{1}=\frac{1}{\lambda_{E}}\int_{0}^{\frac{\alpha_{2}-\psi_{2}\alpha_{1}}{\varphi_{2}\alpha_{1}}}\exp\left(-\frac{\varphi_{2}x+\psi_{2}}{\left(\alpha_{2}-\psi_{2}\alpha_{1}-\varphi_{2}\alpha_{1}x\right)\rho_{S}\lambda_{SR1}}-\frac{x}{\lambda_{E}}\right)dx. \end{array}$$

Similarly, we set new variable as $t_{3}=\alpha_{2}-\psi_{2}\alpha_{1}-\varphi_{2}\alpha_{1}x\to x=\frac{\alpha_{2}-\psi_{2}\alpha_{1}-t_{3}}{\varphi_{2}\alpha_{1}}$ then it can be expressed by

(A15) $$\begin{array}{c} Y_{1}=\frac{1}{\varphi_{2}\alpha_{1}\lambda_{E}}\exp\left(\frac{1}{\alpha_{1}\rho_{S}\lambda_{SR1}}-\frac{\alpha_{2}-\psi_{2}\alpha_{1}}{\varphi_{2}\alpha_{1}\lambda_{E}}\right)q\left(t_{3}\right). \end{array}$$

As a result, we have $Y_{2}$

(A16) $$\begin{array}{cc} Y_{2} & =\int_{0}^{\infty }\exp\left(-\frac{\varphi_{2}x+\psi_{2}}{\rho_{R}\lambda_{kD2}}\right)\frac{1}{\lambda_{E}}\exp\left(-\frac{x}{\lambda_{E}}\right)dx\\ & =\frac{\rho_{R}\lambda_{kD2}}{\rho_{R}\lambda_{kD2}+\varphi_{2}\lambda_{E}}\exp\left(-\frac{\psi_{2}}{\rho_{R}\lambda_{kD2}}\right). \end{array}$$

From (A15) and (A16), $R_{2}$ is rewritten as

(A17) $$\begin{array}{c} Q_{2}=\prod_{k=1}^{K}\left(1-\frac{\rho_{R}\lambda_{kD2}}{\left(\rho_{R}\lambda_{kD2}+\varphi_{2}\lambda_{E}\right)\varphi_{2}\alpha_{1}\lambda_{E}}\exp\left(\frac{1}{\alpha_{1}\rho_{S}\lambda_{SR1}}-\frac{\alpha_{2}-\psi_{2}\alpha_{1}}{\varphi_{2}\alpha_{1}\lambda_{E}}-\frac{\psi_{2}}{\rho_{R}\lambda_{kD2}}\right)q\left(t_{3}\right)\right). \end{array}$$

Then, $P_{SOP2}^{NOMA}$ can be achieved that

(A18) $$\begin{array}{cc} P_{SOP2}^{NOMA} & =\left(1-\frac{\rho_{R}\lambda_{D12}}{\left(\rho_{R}\lambda_{D12}+\varphi_{2}\lambda_{E}\right)\varphi_{2}\alpha_{1}\rho_{S}\lambda_{E}}\exp\left(\frac{1}{\alpha_{1}\rho_{S}\lambda_{D1}}-\frac{\alpha_{2}\rho_{S}-\psi_{2}\alpha_{1}\rho_{S}}{\varphi_{2}\alpha_{1}\rho_{S}\lambda_{E}}-\frac{\psi_{2}}{\rho_{R}\lambda_{D12}}\right)q\left(t_{2}\right)\right)\\ & \times \prod_{k=1}^{K}\left(1-\frac{\rho_{R}\lambda_{kD2}}{\left(\rho_{R}\lambda_{kD2}+\varphi_{2}\lambda_{E}\right)\varphi_{2}\alpha_{1}\lambda_{E}}\exp\left(\frac{1}{\alpha_{1}\rho_{S}\lambda_{SR1}}-\frac{\alpha_{2}-\psi_{2}\alpha_{1}}{\varphi_{2}\alpha_{1}\lambda_{E}}-\frac{\psi_{2}}{\rho_{R}\lambda_{kD2}}\right)q\left(t_{3}\right)\right). \end{array}$$

This is end of the proof. ☐
